# Circular RNA HIPK3 aggravates sepsis-induced acute kidney injury via modulating the microRNA-338/forkhead box A1 axis

**DOI:** 10.1080/21655979.2022.2032974

**Published:** 2022-02-11

**Authors:** Hulin Lu, Yan Chen, Xiaoyi Wang, Yong Yang, Min Ding, Fengping Qiu

**Affiliations:** a Department of Nephrology, Huzhou First People’s Hospital, Huzhou, China; b Department of Infectious Disease, Huzhou First People’s Hospital, Huzhou, China

**Keywords:** Sepsis, CircHIPK3, miR-338, FOXA1, acute kidney injury

## Abstract

Circular RNAs (circRNAs) have been extensively studied in various diseases, including sepsis-induced acute kidney injury (AKI). This research intended to elucidate the mechanism of circular RNA HIPK3 (circHIPK3) in sepsis-engendered AKI. Human tubule epithelial cells (HK2) were stimulated with lipopolysaccharide (LPS) to establish a septic AKI cell model. The gene expression levels were evaluated by RT-qPCR. Cell viability, apoptosis, and cell cycle distribution were assessed through CCK-8 and flow cytometry assays. The potential interactions between genes were verified by luciferase reporter and RIP assays. The results displayed that circHIPK3 expression was enhanced in septic AKI patients and LPS-triggered HK2 cells. Moreover, circHIPK3 interference expedited HK2 cell viability and attenuated apoptosis, inflammatory and oxidative damages following LPS stimulation. Furthermore, circHIPK3 functioned as a molecular sponge for miR-338, and forkhead box A1 (FOXA1) was negatively regulated by miR-338. CircHIPK3 aggravated cell injury in LPS-treated HK2 via targeting miR-338, and FOXA1 addition overturned the suppressing impacts of miR-338-3p augmentation on LPS-activated HK2 cell damage. Finally, we demonstrated that circHIPK3 modulated LPS-induced cell damage via the miR-338/FOXA1 axis. In sum, our results elaborated that circHIPK3 knockdown attenuated LPS-triggered HK2 cell injury by regulating FOXA1 expression via interacting with miR-338, suggesting that circHIPK3 might be a potential biomarker and therapeutic target for sepsis-induced AKI patients.

## GRAPHICAL ABSTRACT

**Figure:**
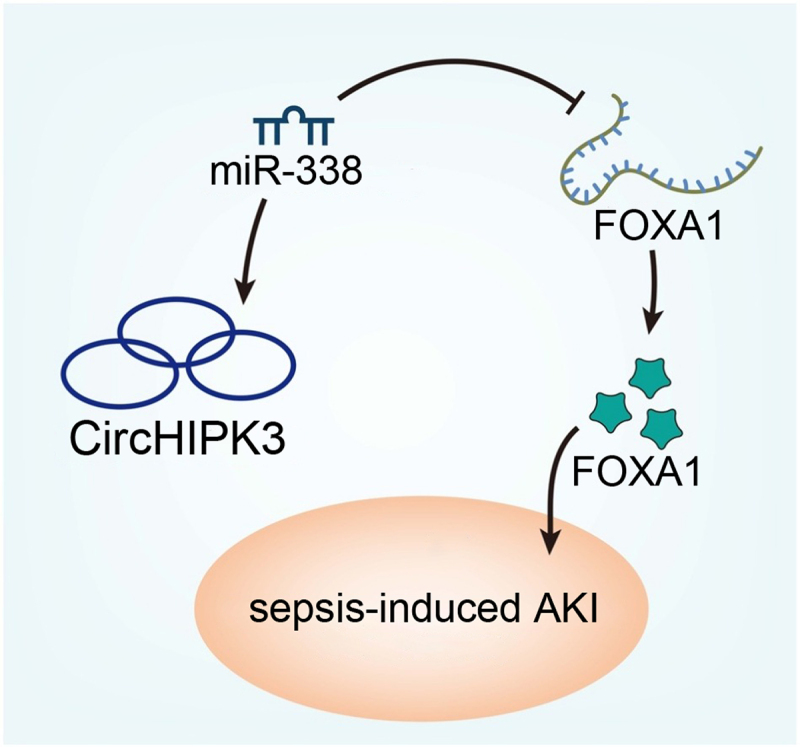

## Introduction

Sepsis is a life-threatening disease characterized by systemic inflammatory response syndrome [1,2]. It is estimated that 18 million new sepsis cases occur each year worldwide with a high fatality rate ranging from 30% to 50% [3,4]. A growing number of researches displayed that sepsis could accelerate the release of inflammatory factors in renal tissues, resulting in renal cell apoptosis, which led to acute kidney injury (AKI) [5,6]. AKI caused by sepsis is closely associated with high mortality of up to 60–80% [7]. Although extensive studies have explored the pathogenesis of sepsis-induced AKI, its pathophysiological mechanisms are still not fully understood [8]. Therefore, providing new insight into the pathogenesis of sepsis-engendered AKI is vital for developing effective therapies for this disease.

Increasing studies have shown that the aberrant expression of circular RNAs (circRNAs) may exert vital biological functions in sepsis. For example, Xiong et al. reported that circ_0003420 mediated lipopolysaccharide (LPS)-induced cell viability and inflammation through decreasing NPAS4 expression in sepsis-associated liver injury [9]. Hong et al. reported that the upregulation of circFADS2 restrained LPS-triggered lung cell apoptosis via repressing miR-15a-5p maturation in sepsis [10]. In addition, the vital function of circRNAs has been identified in AKI. For instance, Shi et al. observed that highly expressed circPRKCI relieved LPS-induced HK2 cell injury through regulating the miR-545/ZEB2 axis [11]. Circ_0114427 facilitated LPS-induced septic AKI by regulating miR-495-3p/TRAF6 via the NF-kappaB pathway [12]. Recently, circular RNA HIPK3 (circHIPK3) has been reported to aggravate cardiac dysfunction in LPS-induced mice models and apoptosis in cardiomyocytes [13]. However, the role and regulatory mechanism of circHIPK3 in sepsis-induced AKI needs further investigation.

In this study, we aimed to investigate the regulatory mechanism of circHIPK3 in sepsis-induced AKI. We assumed that circHIPK3 might regulate forkhead box A1 (FOXA1) expression by sponging miR-338 in sepsis-induced AKI, thus providing a theoretical basis for the treatment of sepsis-induced AKI.

## Materials and methods

### Serum specimens

Human serum samples were recruited from 23 septic AKI patients and 23 healthy subjects at Huzhou first people’s Hospital. This research was approved by the ethics committee of Huzhou first people’s Hospital, and written informed consent was acquired from all participants. The inclusion criteria for patients are as follows: Body temperature (<36°C or >38.5°C), heart rate (> 90 beats/min), tachypnea (>20 breaths/min or PaCO_2_ < 32 mmHg), leukopenia (< 4000/mm^3^), and leukocytosis (> 12,000/mm^3^). The exclusion criteria were the presence of malignancy, pregnancy in female patients, chronic renal insufficiency, use of nephrotoxic drugs, or receiving any kind of renal replacement therapy.

### Cell culture and treatment

Human tubule epithelial cells (HK2) were bought from Cell Bank of the Chinese Academy and kept in DMEM medium (Invitrogen) plus 10% FBS (Procell) and 1% penicillin/streptomycin (Procell) at 37°C with 5% CO_2_.

HK2 cells were treated with different concentrations of LPS (0, 2.5, 5, and 10 µg/mL) for 24 h, and HK2 cells were treated with 5 µg/mL LPS for different times (0, 6, 12, and 24 h). To generate a septic model in vitro, HK2 cells were exposed to 5 µg/mL LPS (Sigma-Aldrich) for 12 h [14].

### Cell transfection

pcDNA3.1 vector expressing circHIPK3 (pcDNA3.1/circHIPK3), pcDNA3.1/FOXA1 and empty pcDNA3.1 vector, small interfering RNA (siRNA) against circHIPK3 (si-circHIPK3) with its control (si-NC), and miR-338 mimics/inhibitor with their controls (NC mimics/inhibitor) were obtained from GenePharma (Shanghai). Lipofectamine 2000 (Invitrogen) was utilized for the transfection.

### CCK-8 assay

HK2 cells (1 × 10^4^ cells/well) were incubated in 96-well plates. Subsequently, 10 μL CCK-8 reagent (Dojindo, Japan) was added and incubated for another 2 h. By using a microplate reader, the absorbance at 450 nm was detected [15].

### RT-qPCR

Total RNA from serums and cells was extracted using the TRIzol reagent (Invitrogen) and then cDNA was synthesized by a PrimeScript RT reagent Kit (Takara, Japan). RT-qPCR was performed with an SYBR Premix Ex Taq II (Takara). Relative levels of genes were evaluated with the 2^−ΔΔCt^ method [16], with U6 or GAPDH as internal references.

### Luciferase reporter assay

The mutant (Mut) or wild-type (WT) sequence of circHIPK3 or FOXA1 was subcloned into pmirGLO vector (Promega) to construct circHIPK3-mut/wt or FOXA1-mut/wt reporters. The vectors were co-transfected with miR-338 mimics or NC mimics into HK2 cells, and the luciferase activity was monitored by Dual-Luciferase Reporter Assay System (Promega) [17].

### Flow cytometry assay

The apoptosis and cell cycle distribution were assessed via flow cytometry. For cell apoptosis, the treated cells were collected, rinsed with PBS, resuspended in 200 μL Annexin V-binding buffer, and then stained with Annexin V-FITC and PI for 15 minutes in darkness. The apoptotic cells were measured by an FCM flow cytometer with FlowJo software (BD Bioscience, USA) [18].

For the examination of cell cycle distribution, transfected HK2 cells were harvested and fixed in 70% ethanol at 4°C. Then, the fixed were treated with RNase in PBS and stained with PI.

### ELISA

The concentrations of IL-1β and TNF-α were evaluated using the corresponding ELISA kits (Beyotime) [19].

### Oxidative stress assay

The contents of malondialdehyde (MDA) and superoxide dismutase (SOD) were determined to assess cell oxidative stress. Briefly, following LPS treatment, the levels of MDA and SOD were examined using the SOD and MDA assay kits (Abcam, Cambridge, UK) [14].

### RIP assay

RIP experiment was tested using the Magna RIP RNA-Binding Protein Immunoprecipitation Kit (Millipore). HK2 cells were lysed in RIP lysis buffer, which was conjugated with anti-Ago2 (Abcam) or anti-IgG conjugated with magnetic beads (Sigma-Aldrich). Immunoprecipitated RNA was purified and tested using RT-qPCR [20].

### Western blot

Proteins were lyzed by RIPA lysis buffer (Beyotime). Then, total protein was separated by 10% SDS-PAGE and transferred onto PVDF membranes (Millipore). Then, the membranes were incubated with primary antibodies against FOXA1 and GAPDH overnight at 4°C, and further incubated with secondary antibodies for 2 h. The bands were evaluated using ECL detection kit (Sigma-Aldrich).

### Statistical analysis

All experiments were performed at least three times, and data were presented as mean ± SD. SPSS 21.0 (IBM Corp.) was adopted for statistical analysis. Student’s t-test or one-way ANOVA followed by Tukey’s test was used to estimate the difference between groups. Correlation between genes was analyzed by Pearson’s correlation analysis. When p < 0.05, there was a statistical difference.

## Results

This work was designed to investigate the function of circHIPK3 in sepsis-induced AKI. Our results demonstrated that circHIPK3 deletion could alleviate LPS-mediated apoptosis, inflammatory, and oxidation stress injuries by regulating FOXA1 expression via targeting miR-338. This study indicated that circHIPK3 might be employed as a potential target for the treatment of sepsis-induced AKI.

### CircHIPK3 expression is elevated in septic AKI

**Figure 1.**
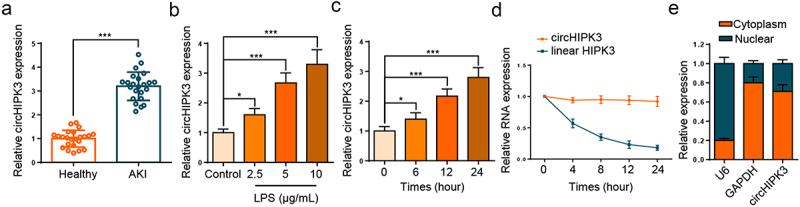
CircHIPK3 expression is elevated in septic AKI patients and LPS-triggered HK2 cells.

Firstly, circHIPK3 expression was assessed in the septic AKI patients, and RT-qPCR experimental data implied that circHIPK3 level was remarkably lifted in serum of septic AKI patients (Figure 1(a), p < 0.001). Next, HK2 cells were stimulated with different LPS concentrations, and RT-qPCR analysis showed that circHIPK3 level was enhanced by LPS treatment in unequal doses (Figure 1(b)). In addition, the expression of circHIPK3 was notably increased in a time-dependent manner (Figure 1(c)). The stability and localization of circHIPK3 were then investigated in HK2 cells. A comparative analysis of circHIPK3 and HIPK3 mRNA expression was performed in HK2 cells that had been treated with actinomycin D. The stability analysis of circHIPK3 and the corresponding linear HIPK3 in HK2 cells showed that the half-life of circHIPK3 was more than 24 h, whereas that of linear HIPK3 was less than 8 h, indicating circHIPK3 was highly stable (Figure 1(d)). In addition, the results of nuclear-cytoplasmic fractionation illustrated that circHIPK3 was predominantly localized in the cytoplasm (Figure 1(e)). These results demonstrated that circHIPK3 was a stable circular RNA and highly expressed in septic AKI.

(a) RT-qPCR showed circHIPK3 expression in serum samples of septic AKI patients and healthy controls. (b) RT-qPCR showed circHIPK3 expression in HK2 cells treated with different concentrations of LPS for 24 h. (c) The expression of circHIPK3 in HK2 cells treated with LPS for different time was detected by RT-qPCR. (d) The stability analysis of circHIPK3 and the linear HIPK3 mRNA in HK2 cells following the treatment of Actinomycin D. (e) Nuclear-cytoplasmic fractionation assay showed that circHIPK3 was mainly located in the cytoplasm. *P < 0.05; ***P < 0.001.

### Interference of circHIPK3 alleviated sepsis-induced AKI in vitro

**Figure 2.**
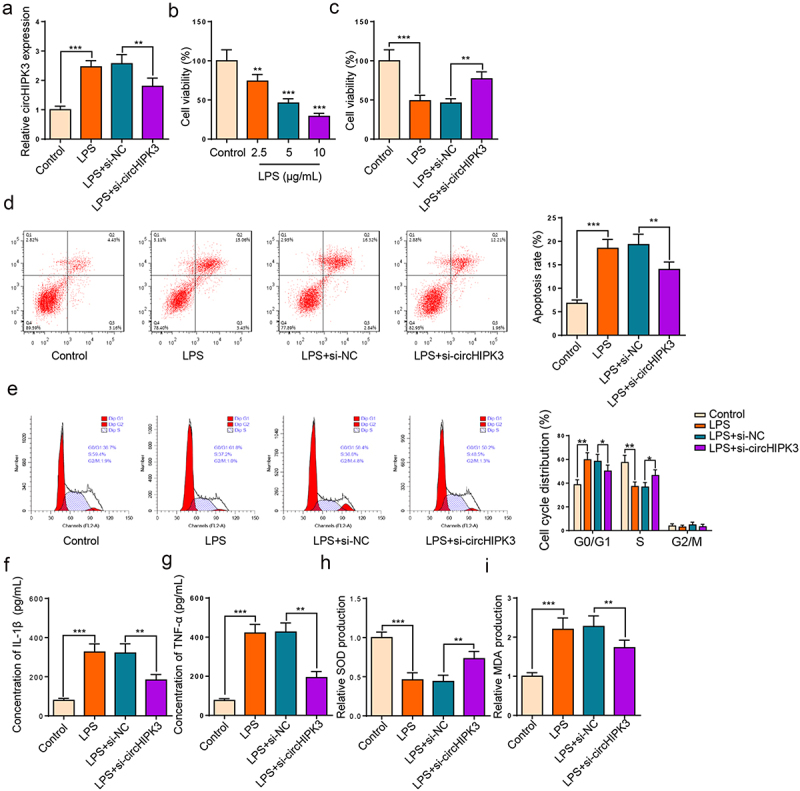
Interference of circHIPK3 expedites viability and suppresses apoptosis and inflammation in LPS-induced HK2 cells.

To determine the function of circHIPK3 in HK2 cells stimulated by LPS, we knocked down circHIPK3. The transfection efficiency of circHIPK3 is presented in Figure 2(a). Then, HK2 cell viability was gradually declined by LPS treatment with the increase of LPS concentration (Figure 2(b)). Moreover, the inhibition of cell proliferation stimulated by LPS was neutralized by circHIPK3 knockdown in HK2 cells (Figure 2(c)). Further, flow cytometry exhibited that HK2 cell apoptosis was accelerated by LPS treatment and cell cycle was arrested at G0/G1 stage; however, circHIPK3 deletion neutralized these impacts (Figure 2(d,e)). In addition, ELISA results unveiled that IL-1β and TNF-α levels were lifted following LPS treatment, which was reversed by circHIPK3 silence (Figure 2(f,g)). Subsequently, we analyzed oxidative indicators to monitor oxidative stress, and the results uncovered that LPS could decline SOD and raise MDA level, suggesting that LPS treatment could promote oxidative stress, while circHIPK3 deficiency offset these effects (Figure 2(h,i)). In sum, these findings suggested that circHIPK3 silence relieved LPS-triggered HK2 cell damage.

(a) RT-qPCR showed circHIPK3 expression in LPS-treated HK2 cells transfected with si-NC or si-circHIPK3. (b) CCK-8 assay showed the viability of HK2 cells treated with different concentrations of LPS. (c) CCK-8 assay showed the viability of HK2 cells treated with LPS, LPS + si-NC and LPS + si-circHIPK3. (d and e) Flow cytometry evaluated cell apoptosis and cell cycle distribution in HK2 cells treated with LPS, LPS + si-NC and LPS + si-circHIPK3. (f and g) ELISA showed the levels of IL-1β and TNF-α in HK2 cells treated with LPS, LPS + si-NC and LPS + si-circHIPK3. (h and i) The levels of MDA and SOD in HK2 cells treated with LPS, LPS + si-NC and LPS + si-circHIPK3 were examined using the corresponding kits. **P < 0.01; ***P < 0.001.

### CircHIPK3 targeted miR-338 in HK2 cells

**Figure 3.**
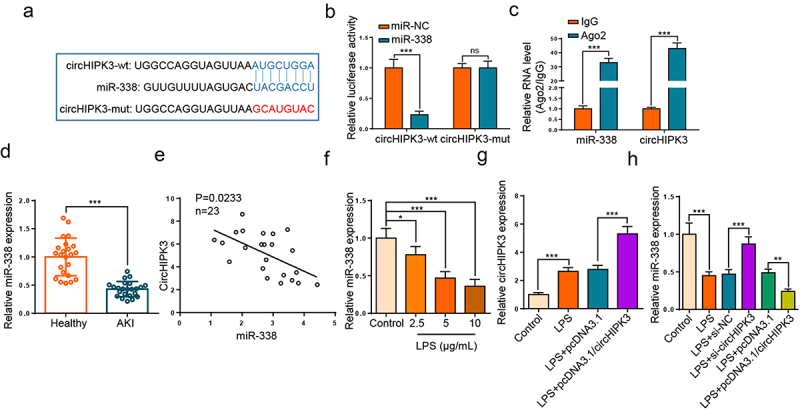
CircHIPK3 targeted miR-338 in HK2 cells.

To find potential targets of circHIPK3, we used circinteractome website (https://circinteractome.nia.nih.gov) to screen the downstream miRNAs with the potential binding ability for circHIPK3. As depicted in Figure 3(a), miR-338 was predicted as a downstream gene of circHIPK3. Moreover, the activity was inhibited in HK2 cells transfected with circHIPK3-wt and miR-338 mimics, while there was no evident impact in the cells with circHIPK3-mut (Figure 3(b)). RIP experiment elaborated that circHIPK3 and miR-338 were enriched in anti-Ago2 group of HK2 cells (Figure 3(c)). Thereafter, RT-qPCR assay elaborated that miR-338 level was downregulated in serum of septic AKI patients (Figure 3(d), p < 0.001). Meanwhile, we found that circHIPK3 level was negatively related to miR-338 (Figure 3(e)). Moreover, LPS treatment repressed miR-338 level in a dose-dependent manner (Figure 3(f)). Furthermore, circHIPK3 addition aggravated the elevated circHIPK3 level induced by LPS (Figure 3(g)). In addition, circHIPK3 silence elevated miR-338 level, while circHIPK3 augmentation decreased miR-338 level in LPS-activated HK2 cells (Figure 3(h)). Collectively, circHIPK3 bound to and negatively modulated miR-338.

(a) The binding sites of miR-338 and circHIPK3 were predicted by circinteractome database. (b) Luciferase reporter assay showed the luciferase activity of circHIPK3-wt/mut in HK2 cells transfected with NC mimics or miR-338 mimics. (c) RIP assay was used to verify the interaction between miR-338 and circHIPK3. (d) RT-qPCR showed miR-338 expression in the serums of septic AKI patients. (e) Pearson’s correlation analysis showed the correlation between miR-338 and circHIPK3 in the serums of septic AKI patients. (f) RT-qPCR showed the level of miR-338 in LPS-stimulated HK2 cells. (g) RT-qPCR showed the expression of circHIPK3 in LPS-induced HK2 cells transfected with pcDNA3.1/circHIPK3 or pcDNA3.1. (h) RT-qPCR showed the level of miR-338 in LPS-induced HK2 cells following the transfection of si-NC, si-circHIPK3, pcDNA3.1 or pcDNA3.1/circHIPK3. *P < 0.05; **P < 0.01; ***P < 0.001.

### miR-338 suppression rescues the impacts of circHIPK3 interference on LPS-engendered HK2 cell damage

**Figure 4.**
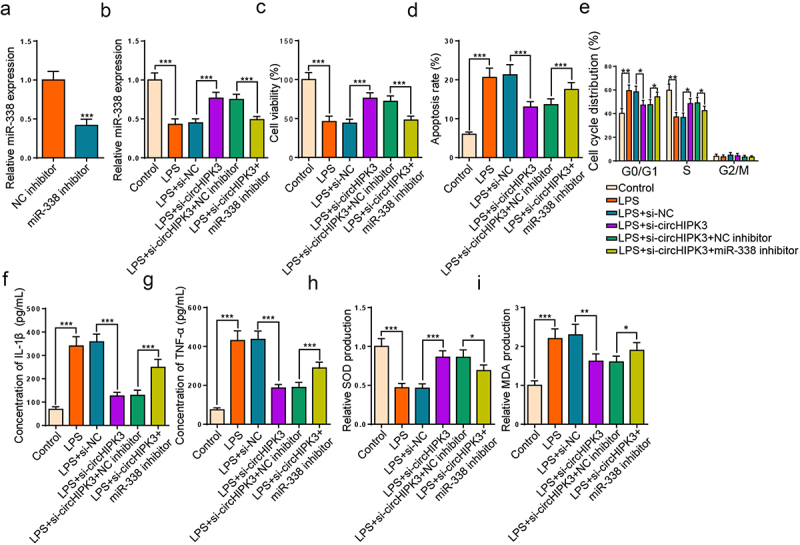
miR-338 suppression rescues the impacts of circHIPK3 interference on LPS-engendered HK2 cell damage.

To further investigate whether circHIPK3 regulated LPS-caused HK-2 cell injury via targeting miR-338, si-circHIPK3 or si-circHIPK3+ miR-338 inhibitor was introduced into LPS-triggered HK-2 cells. As depicted in Figure 4(a), miR-338 level was drastically curbed after its repression. Meanwhile, circHIPK3 repression enhanced miR-338 level, which was declined by miR-338 blocking in LPS-treated HK2 cells (Figure 4(b)). Functionally, circHIPK3 deletion restored LPS-caused the changes of cell viability and apoptosis, while these changes were reversed by miR-338 silence (Figure 4(c,d)). Meanwhile, circHIPK3 deficiency could restrain cell cycle progress in HK2 cells stimulated by LPS, which was mitigated by miR-338 reduction (Figure 4(e)). Further, ELISA assay elaborated that the diminution of IL-1β and TNF-α levels induced by circHIPK3 deletion in LPS-triggered HK2 cells was assuaged by miR-338 deletion (Figure 4(f,g)). Additionally, miR-338 repression neutralized the impacts of circHIPK3 interference on SOD and MDA levels (Figure 4(h,i)). As a result, circHIPK3 interference relieved LPS-activated HK2 cell injury via miR-338.

(a) RT-qPCR was utilized to detect the expression of miR-338 in HK2 cells transfected with NC inhibitor and miR-338 inhibitor. (b) RT-qPCR showed miR-338 expression in HK2 cells treated with LPS, LPS + si-NC, LPS+si-circHIPK3, LPS + si-circHIPK3 + miR-338 inhibitor. (c and d) CCK-8 assay and flow cytometry showed cell viability and apoptosis of HK2 cells treated with LPS, LPS+ si-NC, LPS+si-circHIPK3, LPS+ si-circHIPK3 + miR-338 inhibitor. (e) Flow cytometry showed cell cycle distribution of HK2 cells treated with LPS, LPS + si-NC, LPS+si-circHIPK3 and LPS+ si-circHIPK3 + miR-338 inhibitor. (f and g) ELISA showed the levels of IL-1β and TNF-α in HK2 cells treated with LPS, LPS+ si-NC, LPS + si-circHIPK3 and LPS + si-circHIPK3 + miR-338 inhibitor. (h and i) The levels of MDA and SOD in HK2 cells treated with LPS, LPS + si-NC, LPS + si-circHIPK3 and LPS+ si-circHIPK3 + miR-338 inhibitor using the corresponding kits. *P < 0.05; **P < 0.01; ***P < 0.001.

### FOXA1 directly target miR-338

**Figure 5.**
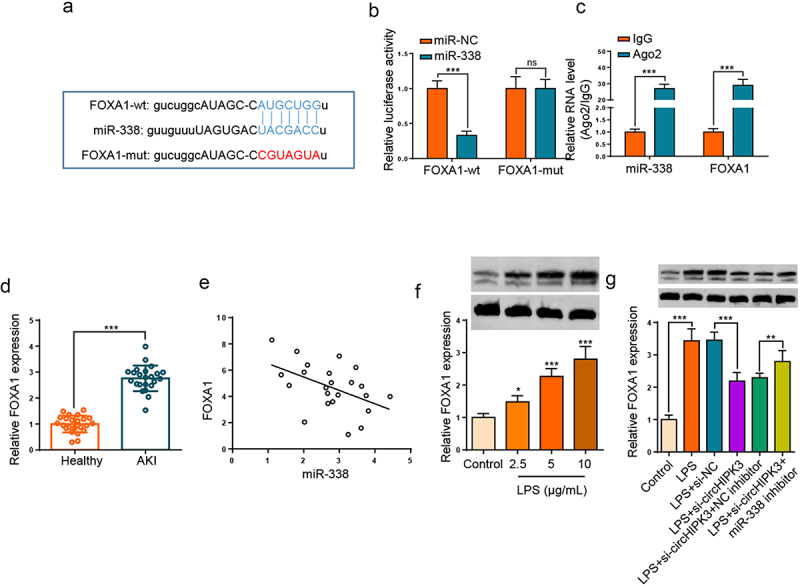
FOXA1 directly target miR-338.

Through searching starBase website, FOXA1 was considered to bind with miR-338 (Figure 5(a)). Then, we manifested that the activity of FOXA1-wt was reduced by miR-338 addition, but had no effects on that of FOXA1-mut (Figure 5(b)). Moreover, RIP experiment confirmed that FOXA1 and miR-338 were enriched in Ago2 complexes (Figure 5(c)). Furthermore, the upregulation of FOXA1 was noticed in the serums of septic AKI patients (Figure 5(d), p < 0.001). As expected, FOXA1 level was inversely associated with miR-338 in sepsis patients (Figure 5(e)). In addition, Western blotting demonstrated that LPS stimulation enhanced FOXA1 level in HK2 cells (Figure 5(f)). In addition, the level of FOXA1 was declined following circHIPK3 deletion in LPS-triggered HK2 cells, however, the impact was restored by miR-338 repression (Figure 5(g)). The aforementioned findings implied that miR-338 inversely modulated FOXA1 level by direct interaction.

(a) The binding sites between miR-338 and FOXA1 were predicted using starBase. (b) Luciferase reporter assay validated the association between miR-338 and FOXA1. (c) RIP assay verified FOXA1 could interact with miR-338. (d) RT-qPCR showed FOXA1 expression in the serums of septic AKI patients. (e) Pearson’s correlation analysis showed the correlation between miR-338 and FOXA1 in the serums of septic AKI patients. (f) Western blotting showed the protein level of FOXA1 in HK2 cells treated with different concentrations of LPS. (g) Western blotting showed the protein level of FOXA1 in HK2 cells treated with LPS, LPS + si-NC and LPS + si-circHIPK3, LPS+ si-circHIPK3 + miR-338 inhibitor. *P < 0.05; ***P < 0.001.

### miR-338 augmentation alleviates LPS-resulted HK2 cell injury via FOXA1

**Figure 6.**
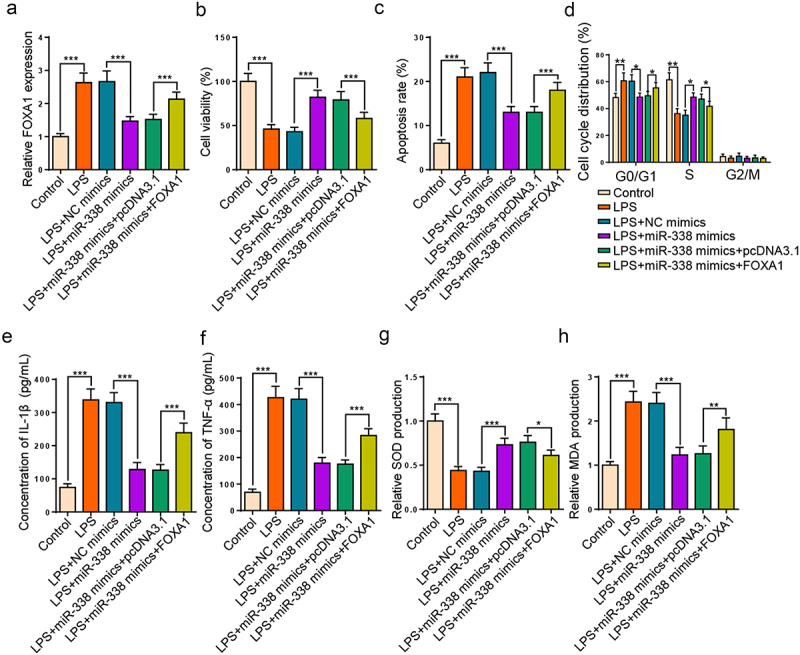
miR-338 augmentation alleviates LPS-resulted HK2 cell injury via FOXA1.

Then, we further investigated whether the regulatory role of miR-338 in LPS-triggered HK2 cell damage could be mediated by regulating FOXA1. RT-qPCR manifested that augmentation of miR-338 declined FOXA1 level in LPS-triggered HK2 cells, while FOXA1 augmentation over-turned this effect (Figure 6(a)). Functional assays unveiled that the miR-338 addition boosted cell proliferation, cell cycle progression, and impeded apoptosis and inflammatory factor levels were counteracted by enhancing FOXA1 level in LPS-triggered HK2 cells (Figure 6(b-f)). Additionally, FOXA1 addition offset the changes of miR-338 supplementation on SOD and MDA levels (Figure 6(g,h)). Collectively, results verified that miR-338 elevation attenuated LPS-injured HK2 cell damage through modulating FOXA1.

(a) RT-qPCR showed FOXA1 expression in HK2 cells treated with LPS, LPS + NC mimics, LPS + miR-338 mimics and LPS + miR-338 mimics + pcDNA3.1/FOXA1. (b-f) CCK-8, flow cytometry and ELISA assays showed cell viability, apoptosis, percentage of cells at different stages and concentration of IL-1β and TNF-α in HK2 cells treated with LPS, LPS + NC mimics, LPS + miR-338 mimics and LPS + miR-338 mimics + pcDNA3.1/FOXA1. (g and h) The levels of SOD and MDA in HK2 cells treated with LPS, LPS + NC mimics, LPS + miR-338 mimics and LPS + miR-338 mimics + pcDNA3.1/FOXA1 were examined by corresponding specific kits. *P < 0.05; **P < 0.01; ***P < 0.001.

## Discussion

Sepsis-caused AKI has become a global health concern and can cause shock and death [21]. Multiple evidence indicated that inflammatory response is involved in the development of septic AKI [22,23]. The levels of pro-inflammatory factors such as IL-6, TNF-α, and IL-1β have been proven to be related to the severity of septic AKI [24]. Oxidative stress refers to the imbalance between the production of oxygen-free radicals and the counteracting of its harmful effects by endogenous antioxidants, resulting in irreversible tissue damage [25,26]. Inflammatory factors and oxidative stress can directly facilitate apoptosis, further aggravating sepsis-induced AKI [27]. Herein, we explored the function of circHIPK3 in septic AKI by establishing septic AKI cell model in vitro.

Many literatures displayed that circRNAs were involved in the development of sepsis-triggered AKI. Xu et al. unveiled that circTLK1 regulated inflammation and oxidative stress via targeting miR-106a-5p and modulating HMGB1 level to contribute to sepsis-triggered AKI [28]. Wei et al. reported that circ_0068,888 facilitated the viability and inhibited inflammatory response to improve sepsis-associated AKI via targeting miR-21-5p [29]. Tan et al. revealed that the addition of circ_0091702 boosted cell viability, restrained the inflammation of LPS-caused HK2 cells by modulating the miR-545-3p/THBS2 axis in septic AKI [30]. CircHIPK3 (has_circ 0000284) is a circular RNA derived from the second exon of the HIPK3 gene, it was reported that circHIPK3 was enhanced in several organs, such as renal and lung [31,32], and acted as an essential modulator in oxidative damage and inflammation [33,34]. Consistent with the previous studies, we uncovered the upregulation of circHIPK3 in serums of septic AKI patients and LPS-treated HK2 cells. Moreover, circHIPK3 blocking neutralized the LPS-induced repression of HK2 cell viability and acceleration of apoptosis, inflammatory reaction, and oxidative stress. Therefore, the above data revealed that circHIPK3 deficiency could alleviate LPS-engendered HK2 cell damage.

It has been acknowledged that circRNAs can competitively bind to miRNAs to modulate mRNAs and thus take part in the pathogenesis of various diseases [35]. miR-338 was uncovered to be decreased in LPS-treated bronchial epithelial cells, and regulated by circ_0038467 to participate in LPS-induced inflammatory injury [36]. In addition, miR-338 was also demonstrated to be downregulated in AKI [37]. Herein, we found that circHIPK3 could bind to miR-338, and miR-338 expression was decreased in septic AKI patients. In addition, circHIPK3 was negatively correlated with miR-338 level. To study the regulatory mechanism of circHIPK3/miR-338 axis, functional experiments were conducted and the results showed that miR-338 silencing restored si-circHIPK3-induced effects on HK2 cell injury. Hence, these results demonstrated that circHIPK3 participated in LPS-triggered AKI via targeting miR-338.

FOXA1, a member of FOXA family, has been reported to play a vital role in various pathological processes, including inflammation, cell viability, and apoptosis [38–40]. Moreover, Lu et al. elaborated that FOXA1 expression was lifted in sepsis-induced AKI patients and promoted HK2 cell apoptosis [41]. In this study, we proved that FOXA1 was negatively regulated by miR-338, and FOXA1 level was increased in septic AKI. Functional assays elaborated that FOXA1 augmentation eliminated the effects of miR-338 addition on LPS-disposed HK2 damage. Moreover, circHIPK3 served as a miR-338 sponge to regulate FOXA1 expression. These data revealed the vital function of the circHIPK3/miR-338/FOXA1 axis in sepsis-induced AKI.

## Conclusion

Our study demonstrated that circHIPK3 interference ameliorated apoptosis, inflammatory factors, and oxidative stress via the miR-338/FOXA1 axis in sepsis-induced AKI, suggesting that circHIPK3 might be a potential biomarker and therapeutic target for sepsis-induced AKI patients. However, the present study had several limitations. First, other downstream regulatory mechanisms of circHIPK3 should be further explored. Second, in vivo experiments should be performed to further understand the pathogenesis of sepsis-induced AKI in the future study.

## Research highlights

- CircHIPK3 expression is elevated in septic AKI
- Knockdown of circHIPK3 alleviated sepsis-induced AKI in vitro
- CircHIPK3 interference relieved LPS-activated HK2 cell injury via miR-338
- MiR-338 augmentation alleviates LPS-resulted HK2 cell injury by targeting FOXA1
